# Combination of curcumin and piperine synergistically improves pain-like behaviors in mouse models of pain with no potential CNS side effects

**DOI:** 10.1186/s13020-022-00660-1

**Published:** 2022-10-23

**Authors:** Pawana Boonrueng, Peththa Wadu Dasuni Wasana, Hasriadi, Opa Vajragupta, Pornchai Rojsitthisak, Pasarapa Towiwat

**Affiliations:** 1 Inter-Department Program of Pharmacology, Graduate School, Chulalongkorn University, Bangkok, 10330 Thailand; 2 Department of Pharmacology and Physiology, Faculty of Pharmaceutical Sciences, Chulalongkorn University, Bangkok, 10330 Thailand; 3 Pharmaceutical Sciences and Technology Program, Faculty of Pharmaceutical Sciences, Chulalongkorn University, Bangkok, 10330 Thailand; 4 Molecular Probes for Imaging Research Network, Faculty of Pharmaceutical Sciences, Chulalongkorn University, Bangkok, 10330 Thailand; 5 Department of Food and Pharmaceutical Chemistry, Faculty of Pharmaceutical Sciences, Chulalongkorn University, Bangkok, 10330 Thailand; 6 Center of Excellence in Natural Products for Ageing and Chronic Diseases, Chulalongkorn University, Bangkok, 10330 Thailand

**Keywords:** CNS side effects, Curcumin, Formalin test, Piperine, Synergistic interaction, Tail-flick test, Cold plate test

## Abstract

**Background:**

Curcumin and piperine are major bioactive compounds of *Curcuma longa* and *Piper nigrum,* widely consumed as spices and flock medicine. The combinational use of these plants is a common practice in Southeast Asia. Synergism between curcumin and piperine has been found in several animal models but not in periodontal disease and diabetes, and the antinociceptive interaction is still unknown. Hence, the present study aimed to assess the interaction between curcumin and piperine in pain and its potential CNS side effect profile.

**Methods:**

Formalin test and in vitro LPS-stimulated RAW 264.7 macrophage cells were used to assess the synergistic interaction of curcumin and piperine in a mouse model of inflammatory pain. Tail-flick and cold plate tests were applied to determine the antinociceptive synergism between piperine and curcumin. The interaction was determined by applying isobolographic analysis. The potential CNS-side effects of the curcumin and piperine combination were also assessed using LABORAS automated home-cage behavioral analysis.

**Results:**

Curcumin alone dose-dependently improved pain-like behaviors in the formalin, tail-flick, and cold plate tests with the ED_50_ of 71.4, 34.4, and 31.9 mg/kg, respectively. Additionally, piperine exhibited efficacy in the formalin, tail-flick, and cold plate tests with the ED_50_ of 18.4, 8.1, and 28.1 mg/kg, respectively. The combination of curcumin and piperine (1:1 ED_50_ ratio) produced synergistic interaction in the formalin, tail-flick, and cold plate tests as assessed significantly lower experimental ED_50_ values (5.9, 5.2, and 5.5 mg/kg) compared to theoretical ED_50_ values (44.9, 21.3, and 30.0 mg/kg), isobologram analysis, and interaction index values of 0.13, 0.24 and 0.18, respectively. The synergistic interaction of curcumin and piperine was further confirmed by the efficacy of the combination in LPS-stimulated RAW 264.7 macrophage cells. Curcumin and piperine interacted synergistically, reducing proinflammatory mediators. The combination also demonstrated better compatibility profiles with neuronal cells. Furthermore, the curcumin-piperine combination had no effects on mouse spontaneous locomotor behaviors in LABORAS automated home cage monitoring.

**Conclusion:**

Overall, the present study demonstrates strong antinociceptive synergism between curcumin and piperine in mouse models with no potential CNS side effects, suggesting its possible use in clinical trials.

## Background

**Fig. 1:**
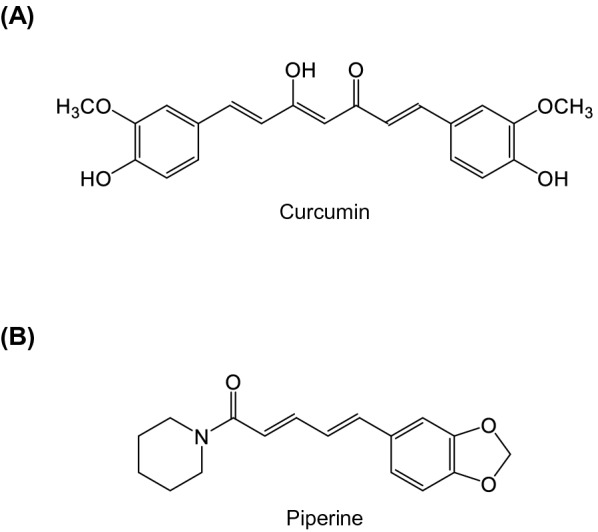
Chemical structures of (**A**) curcumin and (**B**) piperine

For centuries, turmeric containing curcumin and *Piper nigrum* containing piperine have been used as food additives and folk medicine, including Traditional Chinese Medicine (TCM) [1, 2]. Curcumin is a polyphenol compound (Fig. 1A) abundantly found in the *Curcuma longa* Linn. plant [3]. In some countries, average curcumin consumption per day is relatively high due to its use as a cooking spice. In India, 60–100 mg/person/day of curcumin was consumed [4], whereas 2.7–14.8 mg/person/day consumption was found in Korea [5]. In Thailand, turmeric containing curcumin, traditionally called "Khamin Chan," has been used for centuries as a carminative, stomachic, astringent, and coloring agent. In addition, *Curcuma longa* in dry extract and capsule form has been used traditionally to treat joint pain (osteoarthritis) and stomachic [6]. Moreover, curcumin is considered a safe compound and authorized as a GRAS compound (generally recognized as safe) by US FDA (United States Food and Drug Administration) [7]. It is well tolerated at a higher dose of 12 g in humans [7]. Curcumin has also been reported as a potential analgesic both in animals and humans and exhibits diverse cellular and molecular targets [8]. Curcumin inhibits pain neurotransmission by modulating immune and neuronal cells at cellular and mechanistic levels [8, 9]. In animal models of pain, curcumin suppresses pro-inflammatory mediators and increases endogenous anti-inflammatory mediators by modulating peripheral and central immune cells [8, 9]. It also modulates neuronal cells by antagonizing the transient receptor potential vanilloid 1 (TRPV1) ion channels and regulating the expression of purinergic receptors [10, 11]. Despite the efficacy of curcumin as a potential analgesic, poor physicochemical and pharmacokinetic properties remain major challenges that limit its therapeutic use [12]. Hence, numerous approaches have been applied to overcome these limitations, such as nanoformulation, chemical modification, and the combination with other compounds [8]. Several drugs have been combined with curcumin, such as pregabalin, sodium diclofenac, and metformin which produced antinociceptive synergism [13–15]. Their interaction might be from their ability to act on different sites of actions in pain pathways.

Piperine (1-piperoyl piperidine) is a plant alkaloid (Fig. 1B) abundant in *Piper nigrum* Linn. and *Piper longum* Linn. [16]. Black pepper containing piperine, called “Phrik Thai Dam” in Thailand, is commonly used as a food additive and as traditional medicine, such as stomachic and carminative [17]. Piperine is also a bio-enhancer that could improve the pharmacokinetic profiles of other compounds due to its ability to reduce the rate of intestinal and hepatic metabolism [16]. Piperine was found to enhance the pharmacokinetic and pharmacodynamic profiles of ibuprofen [18], ursolic acid [19], and curcumin [20, 21]. Piperine is also reported to have potential analgesic activity via regulating TRPV1, TRPA1, and GABA_A_ receptors [22] and ameliorating the expression of PGE2, IL-6, and MMP13 [23]. Hence, co-administration of piperine with curcumin may potentiate the antinociceptive effects of curcumin as piperine is a bio-enhancer of curcumin, and it has pharmacological efficacy against pain.

The combination of *C. longa* containing a large amount of curcumin, and *P. nigrum* containing a high amount of piperine, has been used in traditional medicine. However, its pharmacological and biological activities have caught recent attention [24, 25]. Many recent studies reported synergistic effects of curcumin and piperine combination in various pharmacological models, including lung cancer [26], aging [27], and hepatocellular carcinoma [28]. Despite the aforementioned favorable effects of combining curcumin and piperine, several studies in murine models of periodontal disease and diabetes failed to prove the synergistic interaction of the curcumin and piperine combination [29, 30]. Therefore, it is necessary to establish the efficacy of curcumin and piperine combination in each pharmacological model, including pain. Hence, in the present study, we investigated the effect of the combination of curcumin and piperine in mouse models of pain, including formalin, tail-flick, and cold plate tests, along with its CNS safety pharmacology. The synergistic interaction at the cellular levels was also investigated in LPS-stimulated RAW 264.7 cells, together with its compatibility with neuronal cells.

## Methods

### Synergism in LPS-induced RAW 264.7 macrophage cells

#### Cell culture

RAW 264.7 macrophage cells were purchased from ATCC (Rockville, MD, USA) and cultured in Dulbecco’s modified Eagle’s medium (DMEM) with 10% fetal bovine serum (FBS) and 1% penicillin–streptomycin (Sigma-Aldrich, MO, USA). The cells were seeded in 24-well plates at the density of 200,000 cells/well in DMEM supplemented with FBS and penicillin-streptomycin and incubated at 37 °C in a 5% CO_2_ atmosphere for 24 h.

#### Cytotoxicity profiling

The cells were treated with different concentrations of curcumin (1.25, 2.5, 5, 10, 20 μM) and piperine (12.5, 25, 50, 100, 200 μM), and the plates were incubated at 37°C in a 5% CO_2_ atmosphere for 24 h. The culture media was removed, and the cells were incubated with a 3-(4, 5-dimethylthiazol-2-yl)-2, 5-diphenyltetrazolium bromide (MTT) solution (0.5 mg/mL) for 2 h. Then, the MTT solution was removed, and dimethyl sulfoxide (DMSO) was added to each well. The absorbance was measured at 570 nm using a microplate reader.

#### NO assay

The NO production in cultured cells was measured using the Griess reaction. The cells were pre-treated with curcumin (0.625, 1.25, 2.5 and 5 μM), piperine (3.125, 6.25, 12.5 and 25 μM) and their combination (0.625 + 3.125, 1.25 + 6.25, 2.5 + 12.5 and 5 + 25 μM Cur + Pip) for 2 h and then challenged with 1 μg/mL LPS for 22 h. Then, 100 μL of cell culture media was transferred to a 96-well plate, followed by adding 50 μL of 1% (w/v) sulfanilamide and incubation in the dark for 5 min. The media was further incubated with 50 μL of 2.5% (w/v) *N*-1-Napthylenediamine dihydrochloride for another 5 min in the dark. The absorbance was measured at 520 nm.

#### Median-effect analysis

The median effect analysis described by Chou-Talalay was employed to determine the type of interaction between curcumin and piperine [31]. The dose–effect relationship between compounds was derived using the median effect equation:

$${\text{F}}_{{\text{a}}} /{\text{F}}_{{\text{u}}} = \, \left[ {{\text{C}}/{\text{C}}_{{\text{m}}} } \right]^{{\text{m}}} ,$$

where F_a,_ fraction effect by compound at the concentration C (F_a_ values ranging from 0 to 1 represent 0 to 100% inhibition of NO production); F_u_, fraction unaffected (F_u_ = 1 – F_a_); C, concentration of test compound; C_m,_ concentration required to produce x% effect; m, sigmoidiciy coefficient of the dose–response curve. Then, the combination index (CI) was determined using the following formula:

$${\text{CI }} = \, \left[ {\text{C}} \right]_{{1}} /\left[ {{\text{C}}_{{\text{x}}} } \right]_{{1}} + \, \left[ {\text{C}} \right]_{{2}} /\left[ {{\text{C}}_{{\text{x}}} } \right]_{{2}} ,$$

where [C]_1_ and [C_2_], compound 1 and 2 concentrations in combination that produce x% effect; [C_x_]_1_ and [C_x_]_2_, compound 1 and 2 concentrations alone that produce x% effect. The interaction between the compounds was further visualized in the fraction affected-combination index (Fa-CI) plot and isobologram. The interaction is identified as an additive, synergistic, or antagonistic if the CI values are 1, < 1, or > 1, respectively.

#### ELISA

The cell culture media was further analyzed using enzyme-linked immunosorbent assay (ELISA) to determine the effect of compounds on LPS-induced proinflammatory cytokine production. IL-6 and TNF-α expression levels in cell culture media were analyzed using a commercial ELISA kit (BioLegend), according to the manufacturer’s instructions.

### Safety evaluation in SH-SY5Y neuronal cells

#### Cell culture

The SH-SY5Y neuroblastoma cells were purchased from ATCC (MD, USA). The cells were maintained in DMEM/F-12 media supplemented with 10% FBS and 1% penicillin-streptomycin and incubated at 37 °C in a 5% CO_2_ atmosphere.

#### Cell viability assay

Cells were seeded in 96-well plates at 50,000 cells/well density and incubated at 37°C in a 5% CO_2_ atmosphere for 24 h. The cells were exposed to EC_50_, EC_75_, and EC_90_ concentrations of curcumin, piperine alone, and their combination (obtained in RAW 264.7 macrophage cell line) for 24 h. Then the cell viability was measured using the MTT assay.

#### Apoptosis and necrosis assay

Hoechst 33342 and Propidium Iodide (PI) staining were used to visualize the morphology and characteristics of apoptotic and necrotic cells, respectively. Cells were seeded in 24-well plates at the density of 200,000 cells/well and treated with EC_90_ concentrations of curcumin, piperine, and their combination for 24 h. Then the cells were washed with PBS and stained with Hoechst 33342 and PI solutions for 15 min. The cell morphology was observed under a fluorescence microscope (Olympus IX51 inverted microscope, Tokyo, Japan), and the images obtained were further processed using Image-J (NIH, MD, USA).

#### Animals

Male ICR mice aged 5–8 weeks (Nomura International, Bangkok, Thailand) were used for all experiments. Mice were acclimatized in the animal facility for at least 1 week before the experiment. The mice were housed 4–5 mice per cage and maintained on 12 h light/dark conditions, with a humidity of 40–60% and a temperature of 23 ± 1 °C with food and water *ad libitum*. In the experiments, animals were randomly selected for a given group. The protocols and procedures were reviewed and approved by the Institutional Animal Care and Use Committee of the Faculty of Pharmaceutical Sciences, Chulalongkorn University (Protocol No. 20–03-003).

#### Compound preparation and administration

Curcumin (> 95.0%) was obtained from Shaanxi Kanglai Ecology Agriculture Co., Ltd., Xi’an, 110 China. Piperine (> 97.0%) was obtained from Sigma, St. Louis, MO, USA. Mice were randomly allocated into five groups for each compound. The behavioral tests were carried out at 09.00 -17.00 in a quiet room during the daytime. On the day of the experiment, mice were allowed to acclimatize 1–2 h to the laboratory room. For curcumin treatment in the formalin test, each group of mice received carboxymethyl cellulose (CMC, 0.5%, in normal saline) and curcumin at 10, 30, 100, 300 mg/kg body weight orally. For piperine treatment in the formalin test, 3, 10, 30, and 100 mg/kg doses of piperine were selected. In a thermal nociceptive test by tail-flick, 3, 10, 30, 100 mg/kg of curcumin and 1, 3, 10, 30 mg/kg of piperine were administered orally. For the cold plate test, curcumin and piperine at 3, 10, 30, 100 mg/kg doses were used. The dose ranges of curcumin and piperine alone were selected according to previous studies [14, 32]. Furthermore, the coadministration of curcumin and piperine to the mice was performed using at least four doses of the combination in a fixed ratio (1:1) of ED_50_ of each treatment alone: 1/2, 1/4, 1/8, and 1/16 × (curcumin ED_50_ + piperine ED_50_). All drugs were suspended in 0.5% CMC and administered orally in a constant volume of 10 ml/kg bodyweight.

### Assessment of pain-like behaviors

#### Formalin test

The subplantar surface of the left hind paw was subcutaneously administered with 10 µL of 5% formalin diluted in normal saline one hour after compound administrations. Duration of licking behaviors as a representative of pain-like behaviors was recorded for 40 min as previously described [33]. The licking behaviors were categorized to phase I (0–5 min) and phase II (10–40 min) for analysis. The percentage antinociceptive efficacy of the test compounds was calculated using the following formula:

$$\% {\text{ antinociception }} = 100{\mkern 1mu} - {\mkern 1mu} \left[ {\left( {{\text{D}}_{{{\text{treatment}}}} /{\text{ D}}_{{{\text{control}}}} } \right){\text{ }} \times {\text{ 1}}00} \right]$$

D_treatment_ is the duration of licking behaviors of mice receiving either monotherapy of curcumin, piperine, or their combination, whereas D_control_ represents the duration of licking behaviors of mice receiving 0.5% CMC.

#### Biochemical analysis of paw tissues and spinal cord

After behavioral measures, mice were euthanized by CO_2_ aspiration, and ipsilateral paw and spinal cord tissues were extracted. Isolated tissues were weighed and mixed with ice-cold PBS (20%, w/v), centrifuged at 10,000 rpm, 4°C for 10 min. Supernatants were collected and stored at -80°C until used for ELISA. IL-6 and TNF-α expression levels in tissue supernatants were analyzed using a commercial ELISA kit (BioLegend, San Diego, CA, USA), according to the manufacturer’s instructions.

#### Tail-flick test

The tail-flick test was selected to assess the effects of the test compounds on thermal/heat nociception. The thermal stimuli from the tail-flick apparatus (Harvard Apparatus, Massachusetts, USA) were applied to the tail of the mice at the mid-region of the dorsal surface. The heat lamp intensity was adjusted to obtain the baseline latency of 3–4 s. The duration of the stimulation until the flicking of the tail was considered tail-flick latency. The cut-off value was set to be 8 s to avoid tail tissue damage. The effects of the test compounds were assessed at time intervals of 0, 15, 30, 60, 90, 120, and 240 min post-compound administration. Percentage antinociception was presented as the percentage of the maximal possible effect of the treatment (%MPE), which was determined using the following formula:

$$\% {\text{MPE }} = {\text{ }}\left[ {\left( {{\text{post-treatment latency }} - {\text{ pre-treatment latency}}} \right){\text{ }}/{\text{ }}\left( {{\text{cut-off }} - {\text{ pre-treatment latency}}} \right)} \right]{\text{ }} \times {\text{ 1}}00$$

#### Cold plate test

The cold plate test was used to assess the effects of curcumin and piperine in monotherapy and combination therapy on cold nociception using a cold plate apparatus (Ugo Basile, VA, Italy). The apparatus was set at 2°C constant temperature, and the baseline latencies to pain-like behaviors (licking, lifting, or shaking of hind paws or jumping out from the cold surface) before compound administration was recorded in triplicate. Then the mice were orally administered with test compounds, and the cold plate latencies were measured 60 min post-compound administration. A cut-off time of 60 s was established to avoid tissue damage. Percentage antinociception was presented as the percentage of the maximal possible effect of the treatment (%MPE), which was determined using the following formula:

$$\% {\text{MPE }} = \, \left[ {\left( {{\text{post-treatment latency }} - {\text{ pre-treatment latency}}} \right) \, / \, \left( {{\text{cut-off }} - {\text{ pre-treatment latency}}} \right)} \right] \, \times { 1}00$$

#### Assessment of CNS safety profile—LABORAS automated home cage behavioral analysis

The effects of the test compounds on spontaneous locomotor activity were assessed in the LABORAS automated home cage behavioral analysis as previously described [34]. Mice were administered with the highest dose of curcumin (300 mg/kg), piperine (100 mg/kg), and their theoretical ED_50_ doses in the formalin test and tail-flick test (44.9 and 21.3 mg/kg, respectively). The spontaneous locomotor activity was measured at one hour post-compound administration for 30 min. The effects of individual curcumin, piperine, and the combination on spontaneous locomotor activity were presented as duration and frequency of mobile behaviors (climbing, rearing, locomotion), immobility, speed, and distance traveled. The position distribution of mice in the cage was also visualized.

### Data analysis

#### ED_50_ analysis

The doses that produce 50% antinociceptive effects in formalin, tail-flick, and cold plate tests were further analyzed. For the individual compound and their coadministration, experimental ED_50_ was determined by linear regression analysis of the log dose–response curve.

#### Isobolographic analysis

Isobolographic analysis was performed to determine the interaction between curcumin and piperine in the formalin, tail-flick, and cold plate tests, as previously described by Tallarida [35]. The experimental ED_50_ and theoretical ED_50_ were determined. The theoretical ED_50_ is calculated using the following formula:

$${\text{ED}}_{{50{\text{add}}}} = {\text{f}}\left( {{\text{ED}}_{{50{\text{D}}1}} } \right) + \left( {1 - {\text{f}}} \right)\,\,\left( {{\text{ED}}_{{50{\text{D}}1}} } \right)$$

where ED_50 add_ represents theoretical ED_50_, ED_50 D1_ represents ED_50_ of curcumin, ED_50 D2_ represents ED_50_ of piperine, f represents fraction.

The isobologram was constructed using the ED_50_ data, and the theoretical ED_50_ of curcumin and piperine were connected using a line (additive line). Further, the experimental ED_50_ of the combination was also included in the isobologram presented as a point. The location of the experimental ED_50_ of the combination in the isobologram was used to determine the antinociceptive interaction between curcumin and piperine. If the point is below the additive line, the interaction is considered synergistic, whereas if the point lies above the additive line, the interaction is considered antagonistic. The significant difference between the theoretical and experimental ED_50_ of the combination was also assessed by t-test to further confirm the antinociceptive interaction. Furthermore, the interaction index was calculated using the following formula:

$$\gamma = {{{\text{ED}}_{50\,\,\exp } \,} \mathord{\left/ {\vphantom {{{\text{ED}}_{50\,\,\exp } \,} {{\text{ED}}}}} \right. \kern-\nulldelimiterspace} {{\text{ED}}}}_{{50\,\,{\text{add}}}}$$

where γ represents the interaction index, ED_50 exp_ represents experimental ED_50_, and ED_50 add_ represents theoretical ED_50_. The interaction index values of < 1, 1, and > 1 are used to define synergistic, additive, and antagonistic interaction, respectively.

### Statistical analysis

All data are presented as means ± SEM. Data were analyzed using GraphPad Prism 9.4.1 by analysis of variance (ANOVA) followed by Bonferroni post hoc test and t-test. The significant level is p < 0.05.

## Results

### Curcumin and piperine alone dose-dependently reduce pain-like behaviors in the mouse formalin model

**Fig. 2:**
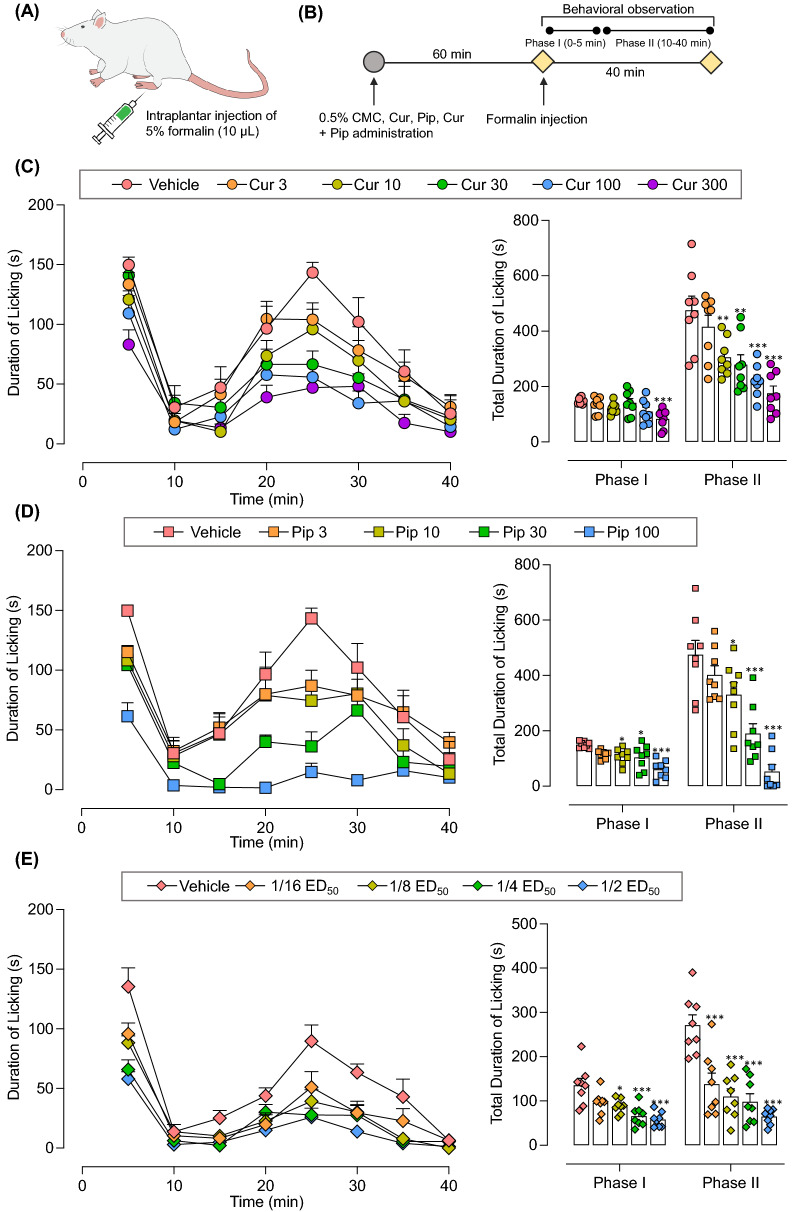
Effect of individual curcumin, piperine, and curcumin-piperine combination on pain-like behaviors in the mouse formalin model. Formalin-induced pain-like behaviors are expressed in the time course of hind paw licking behaviors and the total duration of licking behaviors in phase I (0–5 min after formalin injection) and phase II (10–40 min after formalin injection). Schematic presentation of the experimental design (**A**, **B**). Hind paw licking durations of mice treated with curcumin (**C**), piperine (**D**), and curcumin-piperine combination (**E**). Data are expressed as mean ± S.E.M (n = 8 mice/group). The differences between the vehicle-treated group and treatment groups were analyzed using one-way ANOVA followed by Dunnett’s post hoc test. ***p < 0.001; **p < 0.01; *p < 0.05. CMC, carboxymethyl cellulose; Cur, curcumin; Pip, piperine. The 1/16, 1/8, ¼ and ½ ED_50_ correspond to 5.6, 11.2, 22.5 and 44.9 mg/kg of curcumin-piperine combination

**Fig. 3:**
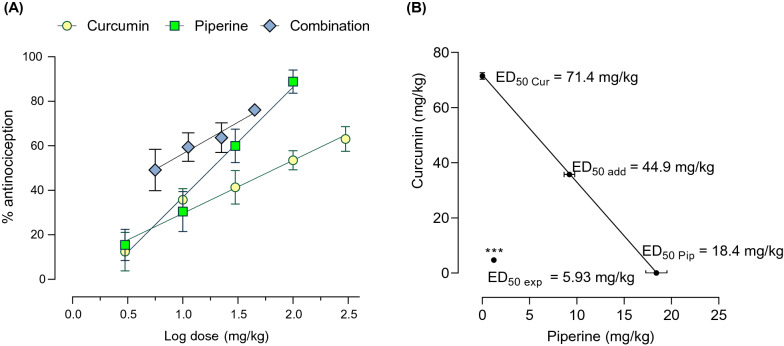
Dose-responses curves of curcumin, piperine, and the combination of curcumin and piperine in the formalin test (**A**) and their isobologram (**B**). Antinociceptive effects are expressed as % antinociception. Data are expressed as means ± S.E.M (n = 8 mice/group). The difference between ED_50 add_ and ED_50 exp_ was analyzed using the unpaired t-test. ***p < 0.001

As shown in Fig. 2, administration of formalin induced biphasic pain-like behavioral response in mice: phase I (0–5 min) and phase II (10–40 min). In phase II, the hind paw licking behavior gradually increased, peaked at 20–25 min, and gradually declined. Both individual administrations of curcumin and piperine attenuated pain-like behaviors in mice induced with formalin in a dose-dependent manner (Fig. 2C and D). When compared with the vehicle group, oral administration of curcumin at doses of 30, 100, and 300 mg/kg and piperine at doses of 10, 30, and 100 mg/kg significantly reduced the duration of licking behaviors in phase II of the formalin test (p < 0.05). The highest dose of curcumin (300 mg/kg) and piperine (100 mg/kg) exerted a 63% and 89% reduction in formalin-induced licking behavior compared to the vehicle-treated group. Not only the phase II, curcumin and piperine also inhibited phase I formalin-induced pain behaviors at higher doses. The individual dose of curcumin and piperine required to exert 50% antinociception in phase II (ED_50_) was then determined using log doses versus % antinociception curves (Fig. 3A). The ED_50_ values of individual curcumin and piperine were determined as 71.4 ± 21.9 and 18.4 ± 3.1 mg/kg, respectively.

### The combination of curcumin and piperine synergistically ameliorates pain-like behaviors in the mouse formalin model

The antinociceptive interaction between curcumin and piperine was determined according to the method established by Tallarida et al*.* [36]. The combinations of curcumin and piperine were orally administered in fixed-dose fractions of their respective ED_50_ of the individual drug in the formalin test (1/2, 1/4, 1/8, and 1/16). ½ ED_50_ dose was 44.9 mg/kg curcumin and piperin combination, containing 35.7 mg/kg of curcumin and 9.2 mg/kg of piperine. As demonstrated in Fig. 2E, co-administration of curcumin and piperine dose-dependently reduced pain-like behaviors in phase II of the formalin test with a maximum antinociceptive effect of 76% at the theoretical additive ED_50_ dose (44.9 mg/kg) (Fig. 3A). The experimental ED_50_ dose was determined at 5.9 mg/kg (4.7 mg/kg of curcumin + 1.2 mg/kg of piperine). Moreover, the dose–response curve for the curcumin and piperine combination shifted left from the dose–response curves of individual treatments (Fig. 3A). Isobolographic analysis of the combination demonstrated the location of the experimental ED_50_ below the predictive additive line, which indicates synergistic interaction between curcumin and piperine in the formalin model (Fig. 3B). Furthermore, statistical analysis confirmed synergistic interaction by the significant difference between theoretical ED_50_ and experimental ED_50_ and the interaction index less than one (0.13) (Fig. 3B, Table 1).

**Table 1:** The antinociceptive activity of individual curcumin, piperine, and curcumin-piperine combination in the formalin, tail-flick and cold plate tests

	ED_50_ ± SEM				γ
	Curcumin	Piperine	Combination		
			Theoretical additive	Experimental	
Formalin Test	71.4 ± 21.9	18.4 ± 3.1	44.9 ± 12.5	5.9 ± 2.2 ^***^	0.13
Tail-Flick Test	34.4 ± 6.1	8.1 ± 0.8	21.3 ± 3.4	5.2 ± 0.6 ^***^	0.24
Cold Plate Test	31.9 ± 5.5	28.1 ± 6.3	30.0 ± 5.9	5.5 ± 0.7	0.18

ED_50_, dose required to exert 50% antinociception; ED_50 add_, theoretical ED_50_; ED_50 exp_, experimental ED_50_; γ, interaction index

### Curcumin and piperine significantly ameliorate formalin-induced peripheral and central inflammation

**Fig. 4:**
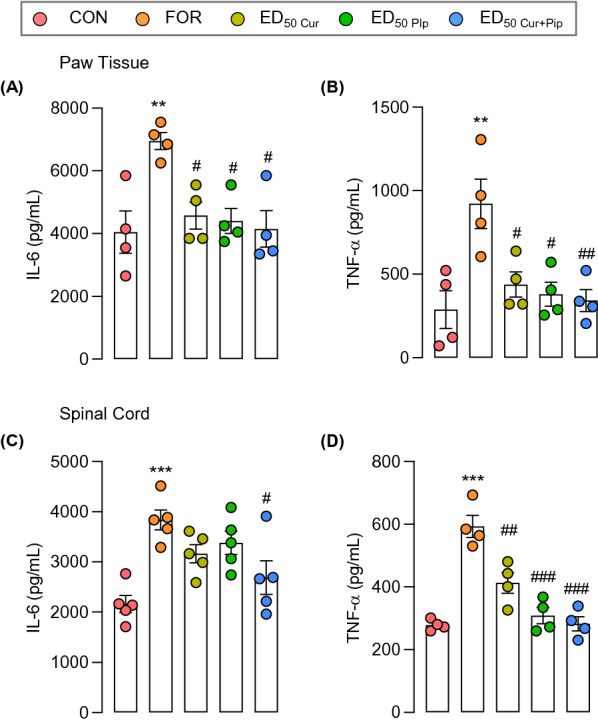
Effect of ED_50_ doses of curcumin, piperine, and curcumin-piperine combination on formalin-induced proinflammatory cytokine production in paw tissues and spinal cord. Effect of each treatment on IL-6 and TNF-α expression in paw tissues (**A** and **B**) and spinal cord (**C** and **D**). Data are expressed as means ± S.E.M (n = 4). The differences between the groups were analyzed using one-way ANOVA followed by the Bonferroni post hoc test. ***p < 0.001, **p < 0.01, compared to the control group and ^###^p < 0.001, ^##^p < 0.01 and ^#^p < 0.05, compared to the formalin group. CON, control group; FOR, formalin; ED_50 Cur_, ED_50 Pip_, and ED_50 Cur+Pip_, ED_50_ doses of curcumin, piperine, and combination, respectively

The proinflammatory cytokine expression in mouse paw tissue and spinal cord samples was evaluated to determine the underline mechanism of curcumin and piperine in inflammatory pain. As demonstrated in Fig. 4, formalin administration significantly increased the proinflammatory cytokine expression (IL-6 and TNF-α) in paw tissues and spinal cords of mice, indicating induction of peripheral and central inflammation, respectively. Treatment with curcumin and piperine alone or in combination at their experimental ED_50_ doses down-regulated the formalin-induced proinflammatory cytokine expression. All treatments showed comparable inhibition in formalin-induced IL-6 and TNF-α production in paw tissue and the spinal cord samples.

### Curcumin and piperine synergistically attenuate inflammatory response in-vitro

**Fig. 5:**
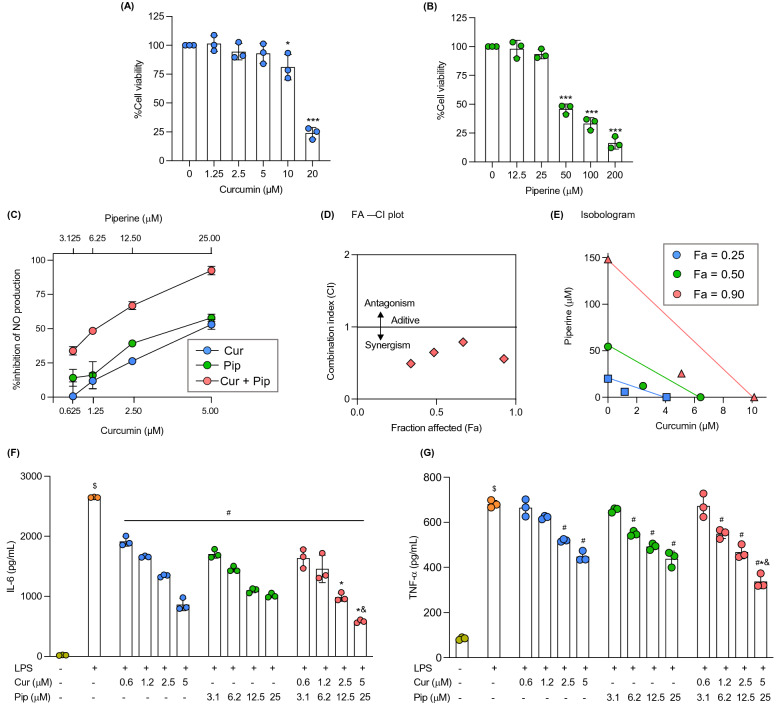
Effect of curcumin and piperine alone and in combination in LPS-induced RAW 264.7 macrophage cells. (**A**, **B**) Cytotoxicity profile of curcumin (**A**) and piperine (**B**) in RAW 264.7 macrophage cells. (**C**) Concentration–response curves for NO inhibitory effects of curcumin, piperine, and combination in LPS-induced RAW 264.7 macrophage cells. (**D**) Fa-CI plot, representing the interaction between curcumin and piperine in NO inhibition. (**E**) Normalized isobologram, representing NO inhibitory effects of curcumin, piperine, and their combination at 25%, 50%, and 90% effect levels. (**F**, **G**) Effect of curcumin, piperine, and their combination on LPS-induced IL-6 (**F**) and TNF-α (**G**) production in RAW 264.7 macrophage cells. Data are expressed as means ± S.E.M (n = 3). The difference between the treatment and control groups was analyzed using ANOVA followed by Dunnett’s post hoc test for cell viability assay. ***p < 0.001, *p < 0.05, compared to the control group. For the cytokine expression, the differences between the groups were analyzed using one-way ANOVA followed by the Bonferroni post hoc test. ^$^p < 0.05, compared to the control group, ^#^p < 0.05, compared to the LPS group. *p < 0.05, compared to the respective individual concentration of curcumin, and ^&^p < 0.05, compared to the respective individual concentration of piperine

The probable interaction between curcumin and piperine at the cellular level was then evaluated using RAW 264.7 macrophage cell line to assess the involvement of peripheral immune cells in the antinociceptive effects observed in phase II of the formalin model. Curcumin and piperine at concentrations higher than 5 and 25 μM, respectively, showed significant cytotoxicity compared to the control (Fig. 5A and B). Hence, in subsequent experiments, curcumin and piperine at the maximum concentration of 5 μM and 25 μM, respectively, were used for combination (1:5 ratio). As shown in Fig. 5C, curcumin, piperine, and their combination inhibited the LPS-induced nitric oxide (NO) production in RAW 264.7 macrophage cells in a concentration-dependent manner. Curcumin-piperine combination exhibited higher %inhibition in NO production compared to the additive effects of curcumin and piperine individual treatment in all concentrations except for the highest concentration. The interaction evaluated by the Chou-Talalay method revealed the synergistic interaction between the compounds at all concentrations tested (Fig. 5D). As indicated in the Fa-CI plot, the CI values at each effect level were lesser than 1, suggesting synergism between compounds. Further, an isobologram was constructed to visualize the interaction between compounds at 25%, 50%, and 90% affect levels (Fig. 5E). As indicated in the isobologram at each affected level, the concentrations of combination required to exert the same effect lie below the respective additivity line, demonstrating synergistic interaction between compounds. For example, the concentrations of curcumin and piperine alone required to inhibit NO production by 50% are 4.1 and 20.0 μM, respectively. However, when the curcumin-piperine combination is used, the ED_50_ dose is reduced to 7.0 μM (1.2 μM Cur + 5.8 μM Pip). Moreover, both curcumin and piperine significantly suppressed the LPS-induced pro-inflammatory cytokine expression (IL-6 and TNF-α) in a concentration-dependent manner (Fig. 5F and G). The curcumin-piperine combination at the highest dose exhibited significantly lesser expression of pro-inflammatory cytokines compared to the individual treatment at respective concentrations.

### Curcumin-piperine combination showed better compatibility with neuronal cells compared to individual treatments

**Fig. 6:**
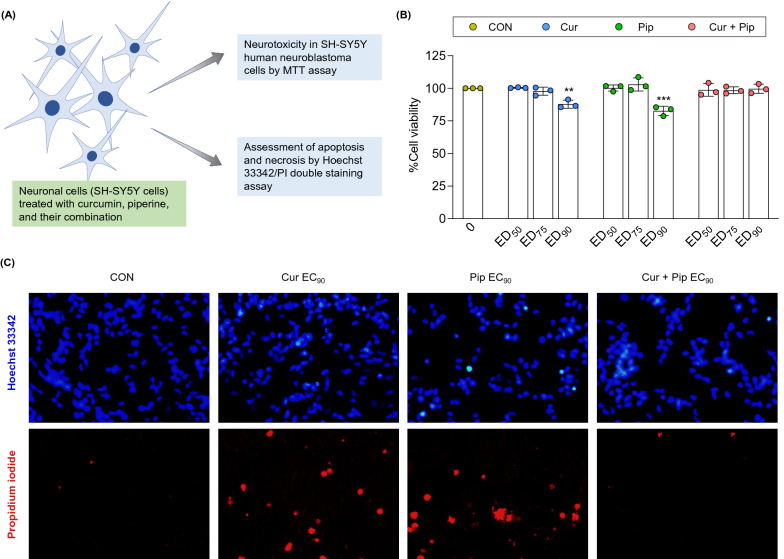
Compatibility of curcumin, piperine, and their combination with SH-SY5Y neuronal cells. (**A**) Experimental design. (**B**) Cell viability after treatment with EC_50_, EC_75_, and EC_90_ concentrations of curcumin (4.0, 6.4, and 10.2 μM), piperine (20.0, 54.5, and 148.3 μM), and curcumin-piperine combination (1.2 + 5.8, 2.4 + 12.2, and 5.1 + 25.5 μM Cur and Pip). (**C**) Effect of curcumin, piperine, and their combination at EC_90_ concentration on apoptosis and necrosis visualized by Hoechst 33342/PI double staining. Data are expressed as means ± S.E.M (n = 3). The differences between the control and treatment groups were analyzed using one-way ANOVA followed by Dunnett’s post hoc test. ***p < 0.001, **p < 0.01

To predict the safety of curcumin-piperine in neuronal cells, the toxicity of curcumin, piperine alone, and in combination at their EC_50_, EC_75_, and EC_90_ concentrations were evaluated in the SH-SY5Y neuroblastoma cell line (Fig. 6A). As shown in Fig. 6B, curcumin and piperine at ED_90_ concentrations (148.3 and 10.2 μM, respectively) significantly reduced the cell viability compared to the control cells. However, the curcumin-piperine combination at the EC_90_ concentration, 30.6 μM (5.1 μM Cur + 25.5 μM Pip), exhibited no toxicity to SH-SY5Y cells. Further, the Hoechst 33342 and PI staining showed apoptosis and necrosis in cells treated with EC_90_ concentrations of curcumin and piperine but less evident in cells treated with the curcumin-piperine combination (Fig. 6C). The cells treated with EC_90_ concentrations of curcumin and piperine alone showed condensed and fragmented nuclei in Hoechst 33342 staining, referring to cellular apoptosis, and evident red color staining in PI staining, referring to necrosis.

### Curcumin and piperine alone dose-dependently reduce pain-like behaviors induced by heat stimuli

**Fig. 7:**
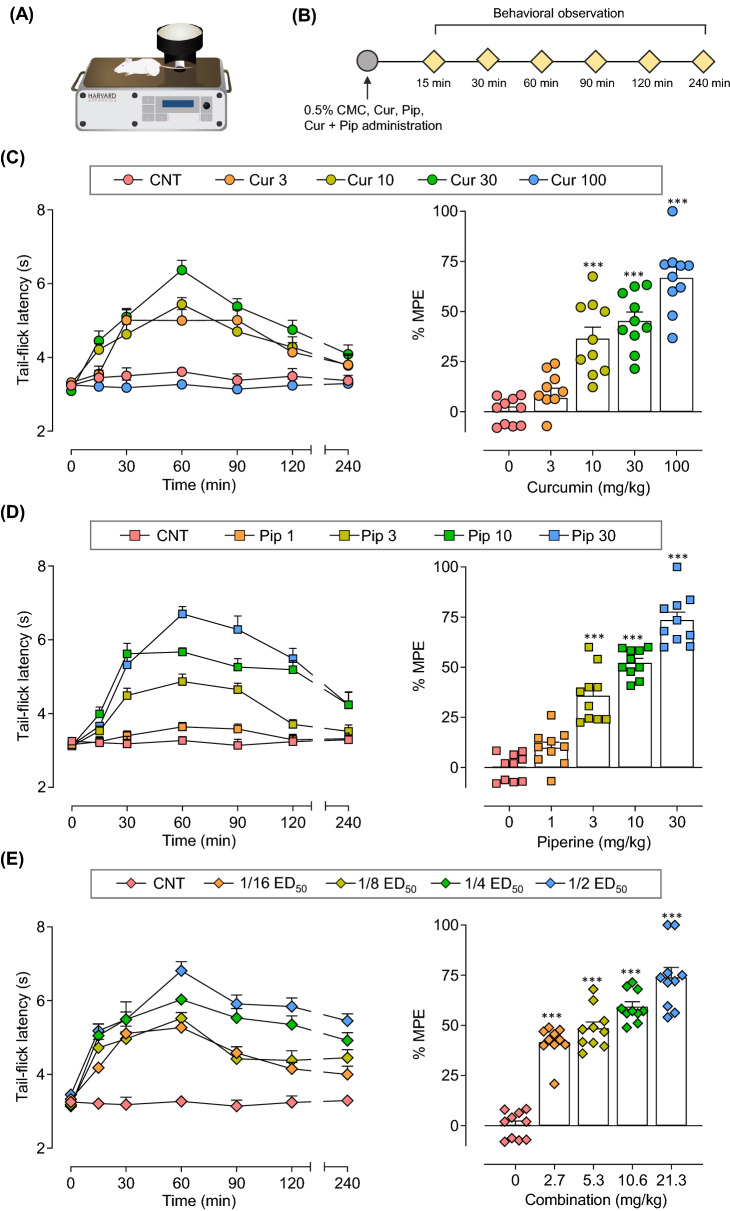
Effect of curcumin, piperine and their combination on heat nociception. (**A**) Experimental setup. (**B**) Experimental timeline. (**C, D, E**) Time course of the effect of curcumin (**C**), piperine (**D**) and the combination of curcumin and piperine (**E**) on thermal nociception in mice. The effects of the test compounds were expressed as the time course of tail-flick latency and % maximum possible effects at 60 min post-compound administration. Data are expressed as means ± S.E.M (n = 10 mice/group). The differences between the vehicle-treated group and treatment groups were analyzed using one-way ANOVA followed by Dunnett’s post hoc test. ***p < 0.001; **p < 0.01; *p < 0.05, compared to the control group

**Fig. 8:**
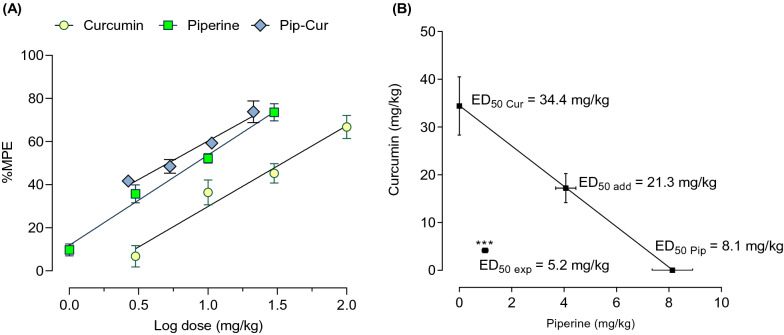
Dose-responses curves of curcumin, piperine, and the combination of curcumin and piperine in the tail-flick test (**A**) and their isobologram (**B**). Antinociceptive effects are expressed as %MPE. Data are expressed as mean ± S.E.M (n = 10 mice/group). The difference between ED_50 add_ and ED_50 exp_ was analyzed using the unpaired t-test. ***p < 0.001

The effects of oral administration of individual curcumin and piperine on acute nociceptive pain were assessed in the tail-flick test. After each compound administration, tail-flick latency was measured at 0, 15, 30, 60, 90, 120, and 240 min post-compound administration. As shown in Fig. 7, the tail-flick latency of vehicle-treated mice remained unchanged (3–4 s) throughout the 240 min experimental period, whereas treatment with individual curcumin and piperine dose-dependently increased the tail-flick latency. Compared with the vehicle-treated group, curcumin at 10, 30, and 100 mg/g doses and piperine at 3, 10, and 30 mg/kg doses significantly attenuated thermal stimuli-induced nociceptive pain in the tail-flick test. The peak effect of either individual curcumin or piperine was observed at 60 min post-compound administration and was used to calculate the antinociceptive effects of the compounds presented as %MPE (Fig. 7C–E). The curcumin and piperine at the highest doses tested effectively attenuated thermal nociception in mice up to 67% and 74%, respectively (Fig. 7C and D). The ED_50_ doses were derived using respective dose–response curves: ED_50_ of curcumin and piperine were 34.4 ± 6.1 and 8.1 ± 0.8, respectively (Fig. 8A).

### The combination of curcumin and piperine synergistically interacts in suppressing pain-like behaviors induced by heat stimuli

After investigating the effects of individual drugs in the tail-flick test, the combination of curcumin and piperine was further tested. As shown in Fig. 7E, the combination of curcumin and piperine dose-dependently improved pain-like behaviors induced by thermal stimuli. The peak effect of the combination was observed at 60 min post-compound administration, which was further used to calculate %MPE. Curcumin-piperine combination at the theoretical ED_50_ dose (21.3 mg/kg) significantly decreased thermal nociception by 74% compared to the vehicle-treated group (Fig. 8A). Moreover, the dose–response curve for the combination shifted left from the individual dose–response curves of curcumin and piperine. Accordingly, the ED_50_ of the combination was 5.2 ± 0.6 mg/kg (4.2 mg/kg curcumin and 1 mg/kg piperine). The administration of curcumin and piperine in combination significantly reduced the ED_50_ dose by 76% from the predicted ED_50_ dose (*p* < 0.001, *t*-test). Furthermore, the location of experimental ED_50_ below the additive line of the isobologram (Fig. 8B) and the interaction index value of 0.24 indicate a strong antinociceptive synergism between curcumin and piperine in the tail-flick test.

### Curcumin and piperine alone dose-dependently reduce pain-like behaviors induced by cold stimuli

**Fig. 9:**
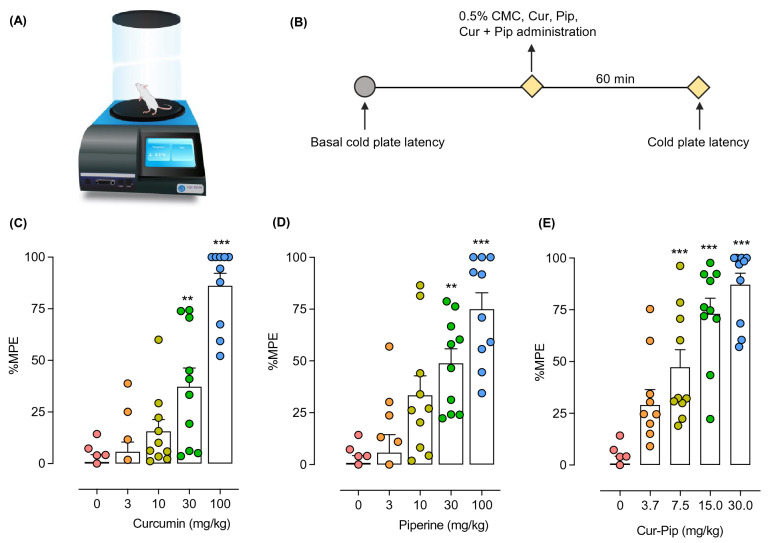
Effect of curcumin, piperine, and their combination on cold nociception in mice. (**A**)Experimental setup. (**B**) Experimental timeline. (**C**, **D**, **E)** The effect of curcumin (**C**), piperine (**D**), and their combination (**E**) on cold nociception is expressed as the %maximum possible effect. Data are expressed as means ± S.E.M (n = 10 mice/group). The differences between the vehicle-treated and treatment groups were analyzed using one-way ANOVA followed by Dunnett’s post hoc test. ***p < 0.001; **p < 0.01, compared to the control group

The efficacy of curcumin, piperine, and their combination in attenuating the cold nociception was evaluated using the cold plate test. As shown in Fig. 9, curcumin and piperine dose-dependently enhanced the cold tolerance in mice compared to the vehicle-treated mice. Curcumin and piperine at 100 mg/kg dose significantly attenuated cold nociception up to 86.1 and 74.9%, respectively. The ED_50_ doses were calculated from respective dose–response curves. Curcumin at 31.92 ± 5.5 mg/kg and piperine at 28.1 ± 6.3 mg/kg exerted 50% antinociception to cold stimuli.

### The combination of curcumin and piperine synergistically interacts in suppressing pain-like behaviors by cold stimuli

**Fig. 10:**
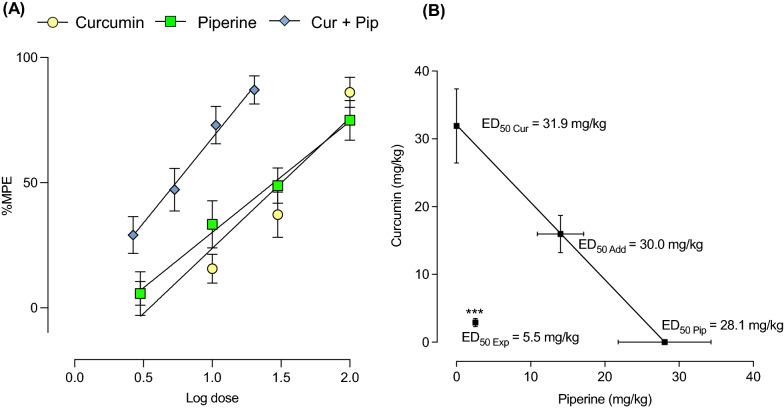
Dose-responses curves of curcumin, piperine, and the combination of curcumin and piperine in the cold plate test (**A**) and their isobologram (**B**). Antinociceptive effects are expressed as %MPE. Data are expressed as mean ± S.E.M (n = 10 mice/group). The difference between ED_50 add_ and ED_50 exp_ was analyzed using the unpaired t-test. ***p < 0.001

Following the evaluation of curcumin and piperine alone in the cold plate test, the combination of them at the ratio of their ED_50_ doses was evaluated. Curcumin and piperine combination dose-dependently improved pain-like behaviors induced by cold stimuli (Fig. 9E). Curcumin and piperine at their theoretical ED_50_ dose (30.0 mg/kg) exerted 87.0% antinociception compared to the vehicle-treated group. The dose–response curve for the combination shifted left from the individual-dose response curves (Fig. 10A), and the experimentally derived ED_50_ dose for the combination was 5.5 ± 0.7 mg/kg (2.9 mg/kg Cur + 2.6 mg/kg Pip). The isobologram analysis further revealed the location of the experimental ED_50_ value below the additive line (Fig. 10B), and the interaction index was 0.18, indicating a strong antinociceptive synergism between compounds to the cold stimuli.

### No effects of the combination of curcumin and piperine on spontaneous locomotor activity

**Fig. 11:**
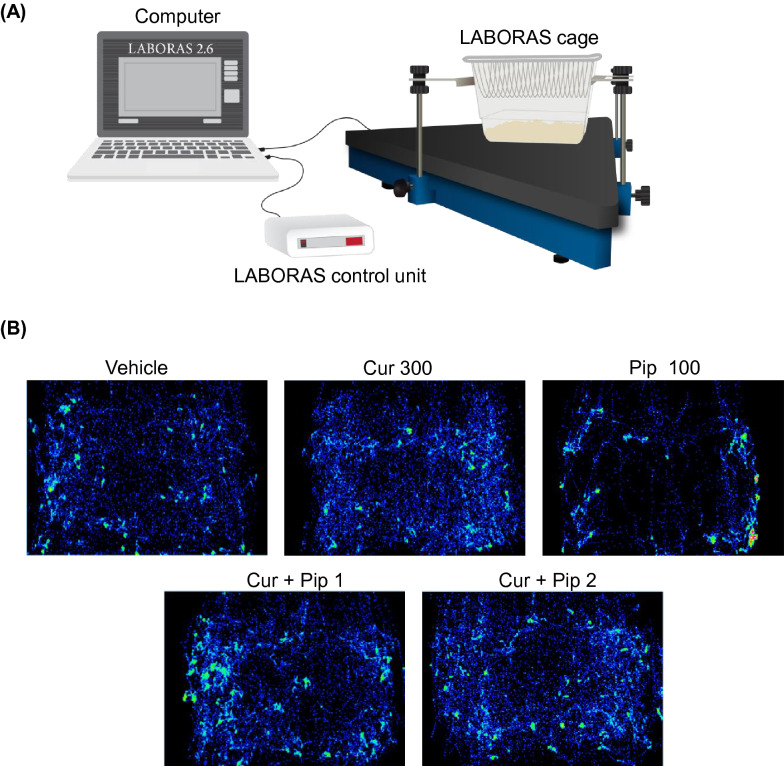
Experimental setup of LABORAS (**A**) and traces of the mice on the cage after treatment of vehicle, curcumin, piperine, and combination of curcumin and piperine. Cur300, curcumin 300 mg/kg; Pip100, piperine 100 mg/kg; Cur + Pip1; theoretical ED_50_ dose in the formalin test; Cur + Pip2; theoretical ED_50_ dose in the tail-flick test

**Fig. 12:**
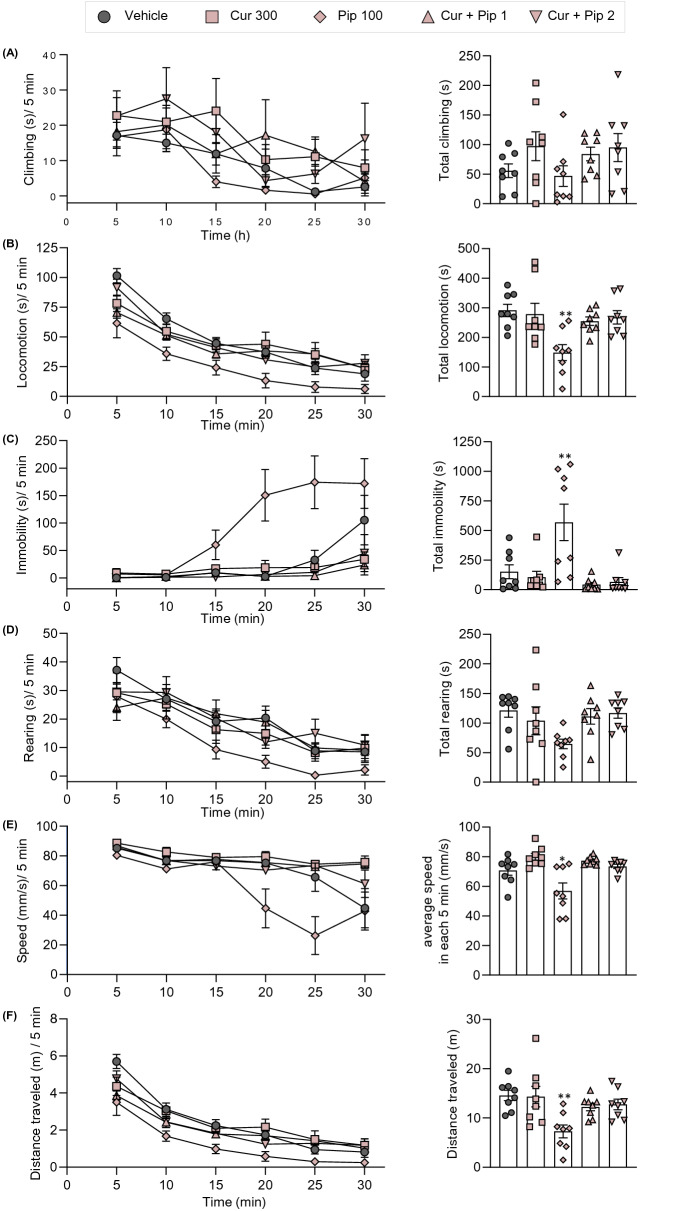
The effects of curcumin, piperine, and the combination of curcumin and piperine on spontaneous locomotor activity in naïve mice. The behaviors were presented as the duration of climbing (**A**), locomotion (**B**), immobility (**C**), rearing (**D**), average speed (**E**), and distance traveled (**F**). Data are expressed as means ± S.E.M (n = 8 mice/group). Cur300, curcumin 300 mg/kg; Pip100, piperine 100 mg/kg; Cur + Pip1; theoretical ED_50_ dose in the formalin test; Cur + Pip2; theoretical ED_50_ dose in the tail-flick test

**Fig. 13:**
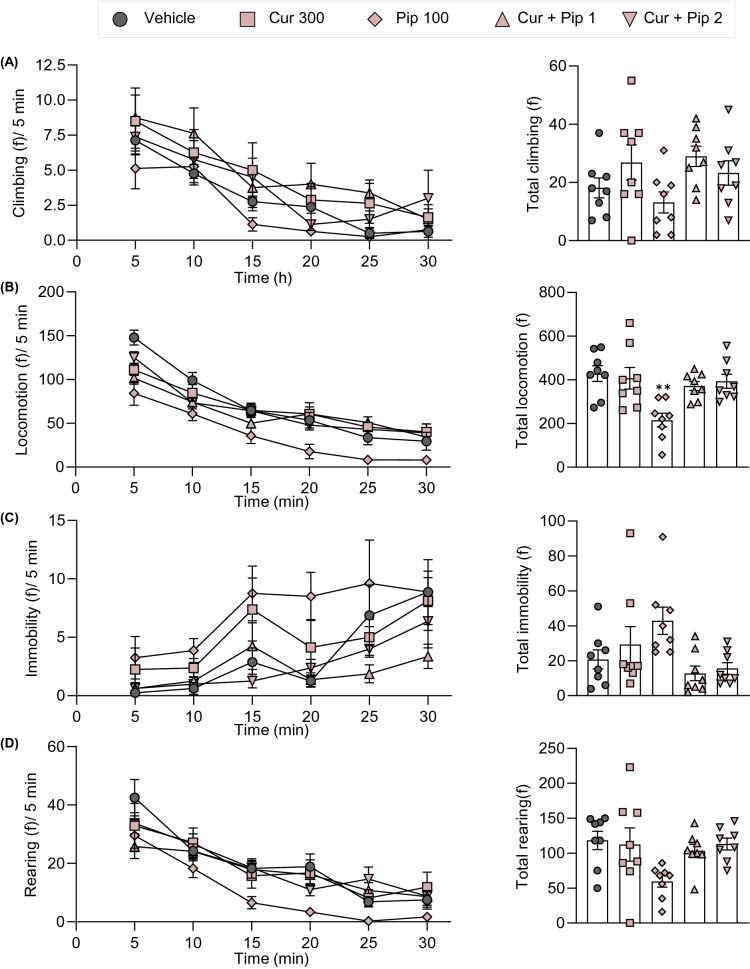
The effects of curcumin, piperine, and the combination of curcumin and piperine on spontaneous locomotor activity in naïve mice. The behaviors were presented as the frequency of climbing (**A**), locomotion (**B**), immobility (**C**), and rearing (**D**). Data are expressed as means ± S.E.M (n = 8 mice/group). Cur300, curcumin 300 mg/kg; Pip100, piperine 100 mg/kg; Cur + Pip1; theoretical ED_50_ dose in the formalin test; Cur + Pip2; theoretical ED_50_ dose in the tail-flick test

To determine the potential side effects of curcumin on CNS, spontaneous locomotor activity after administration of the test compounds was assessed in the LABORAS (Fig. 11A). Mice were administered with the highest dose of curcumin (300 mg/kg), piperine (100 mg/kg), and their theoretical ED_50_ doses in the formalin test and tail-flick test (44.9 and 21.3 mg/kg, respectively). The spontaneous locomotor activity was measured at 1 h post-compound administration for 30 min. The results demonstrated that the administration of the highest dose of curcumin did not affect spontaneous locomotor activity, whereas the administration of the highest dose of piperine impaired locomotor activity. As shown in Fig. 11B, mice treated with vehicle and curcumin explored the entire cage. In contrast, the position distribution of mice treated with piperine was mostly limited to the edges of the cage, indicating impaired exploratory behaviors. The impairment of spontaneous locomotor activity by piperine was also characterized by a reduction in mobile behaviors (climbing, locomotion, and rearing) and increasing immobility. The statistically significant difference in locomotive behaviors between vehicle- and piperine-treated groups was observed in locomotion (s), locomotion (f), immobility (s), speed (mm/s), and distance traveled (m) (Figs. 12 and 13). Interestingly, treatment with curcumin and piperine combination doses showed no effects on locomotive behaviors in mice.

## Discussion

The main objective of the present study was to determine the synergistic interaction between curcumin and piperine in mouse models of pain. The results demonstrated that both curcumin and piperine alone reduced pain-like behaviors in the formalin, tail-flick, and cold plate tests. In addition, the fixed-dose fractions of curcumin and piperine combination produced synergistic interaction in formalin, tail-flick, and cold plate tests in mice. Furthermore, a significant reduction in locomotive behaviors was only observed with the administration of the highest dose of piperine but not with the curcumin or curcumin-piperine combination, indicating no potential CNS side effects of curcumin-piperine combination at its highest therapeutic doses.

In the present study, the formalin-induced mouse model was used as a model of inflammatory pain. In the formalin test, hind paw licking is identified as pain-like behavior and categorized into two phases. Phase I is the direct sensitization of formalin to peripheral neurons, while Phase II is the sensitization of peripheral neurons due to inflammatory response. Moreover, intraplantar administration of formalin causes peripheral immune cells to be recruited and infiltrated into the inflammatory sites [37]. Non-neuronal cells, such as macrophages, are reported to have a role in initiating inflammatory pain by releasing proinflammatory mediators, which then enhance pain neurotransmission [38]. Apart from the short-term responses, phase II is marked by a continuous release of proinflammatory mediators caused by the activation of spinal microglia, which sensitize the projection neurons leading to central sensitization [39–41]. On the other hand, pain-like behaviors induced by thermal stimuli (hot and cold) in the tail-flick and cold plate tests are identified as the withdrawal of the tail from the radiant heat and licking of the hind paw or jumping off against cold stimuli, respectively. These thermal models, thought to be a spinal reflex, might engage higher brain systems, mainly indicating central analgesia [42, 43]. In addition, the tail-flick test is characterized by activation nociceptors, TRPV1 and TRPV3 [44]. For the cold plate, pain-like behaviors are mechanistically initiated by activating TRPA1 [45] and TRPM8 [46]. Therefore, effective attenuation of pain by curcumin, piperine, and their combination in the formalin, tail-flick, and cold plate tests indicates their plausible effects on both peripheral and central sensitization by modulation nociceptors and inflammatory mediators. At the cellular level, using macrophage cells, curcumin and piperine interacted synergistically, suppressing inflammatory mediators with favorable compatibility with neuronal cells. In addition, biochemical analysis of paw tissue and spinal cord samples in mice that underwent formalin test revealed significant attenuation of proinflammatory cytokine expression by curcumin, piperine alone, and in combination in both tissues indicating their potential to alleviate peripheral and central inflammation.

Curcumin and piperine have been shown to interact synergistically in various preclinical pharmacological studies [26–28]. In contrast, curcumin and piperine failed to exhibit synergistic interactions in periodontal disease and diabetic models [29, 30]. Furthermore, the efficacy of lipoic acid plus curcumin phytosome and piperine has been evaluated in humans with neuropathic pain, yet the interaction between those two compounds in pain models remains to be determined [47]. Therefore, controversy still exists as to whether curcumin and piperine combination can synergistically interact in mouse models of pain. In the present study, individual curcumin and piperine significantly reduced pain-like behaviors induced by formalin and thermal stimuli in a dose-dependent manner. Furthermore, the combination of curcumin and piperine elicited a greater antinociceptive effect compared to that of either curcumin or piperine alone. The isobolographic analysis was performed to evaluate the type of interaction, wherein strong antinociceptive synergism between curcumin and piperine was found. This interaction produced by the combination of these two compounds could be due to their pharmacodynamic and pharmacokinetic interactions.

Recently, a growing body of evidence has shown that compounds with different sites of action in pain pathways have a higher potential to elicit synergistic interactions. Curcumin exhibits diverse cellular and molecular actions. It inhibits pro-inflammatory mediator release by activated-peripheral and central immune cells. Furthermore, curcumin also modulates neuronal cells via TRPV1, purinergic, and chemokine receptors. In addition, piperine was also found to improve pain-like behaviors via modulating neuronal receptors, such as TRPV1, TRPA1, and GABA_A_ receptors [22]. Pharmacodynamically, the combination of curcumin and piperine could simultaneously inhibit multiple sites of action in the pain pathway. The ability of curcumin and piperine to modulate diverse pathways of pain could lead to robust inhibition of pain transmission when those are coadministered. Hence, the pharmacodynamic interaction between curcumin and piperine could be one of the potential reasons for the observed antinociceptive synergism in this study.

In addition, it has also been proved that piperine can increase the delivery of curcumin to systemic circulation due to its ability to improve the pharmacokinetic profiles of curcumin. Piperine was reported to increase the bioavailability of oral curcumin: 154% and 2000% increase in curcumin concentration in the plasma was observed in rats and humans, respectively [20, 21]. The increased concentration of curcumin in the bloodstream is due to the ability of piperine to decrease the rate of metabolism of curcumin in the intestine and liver. In the intestine, piperine regulates membrane lipid dynamics and inhibits the intestinal metabolism of curcumin, leading to enhanced curcumin retention in the intestine [48]. Furthermore, piperine reduces the rate of curcumin metabolism in the liver by hindering aryl hydrocarbon hydroxylation, ethylmorphine-N-demethylation, 7-ethoxycoumarin-O-deethylation, and 3-hydroxy-benzo(a)pyrene glucuronidation and glucuronidation [49], which subsequently reduce the first-pass metabolism of curcumin. Hence, coadministration of piperine and curcumin enhances the oral bioavailability of curcumin, and thereby, its therapeutic efficacy. This factor may also have influenced the strong antinociceptive synergism observed in this study.

Reducing the therapeutic dose of curcumin and piperine can potentially minimize their side effects since previous studies showed potential side effects of both curcumin and piperine at higher doses [50]. At a higher dose, curcumin causes diarrhea and nausea [51]. For piperine, it can cause respiratory paralysis and edema in the urinary and gastrointestinal tracts [52]. Therefore, reducing the dose of the compounds will also reduce their side effects which can be achieved by administering drugs in combination form. In the present study, despite its combination efficacy, the potential side effects of the combination were determined. At the cellular level, neuronal cells treated with EC_90_ concentrations of curcumin and piperine showed significant cytotoxicity to neuronal cells. However, the cells treated with the curcumin-piperine combination at their EC_90_ concentration showed no toxicity to neuronal cells. This could be due to the reduced dose requirement in combination therapy, leading to reduced toxicity. Further, the CNS safety profile of the combination was evaluated in the LABORAS by assessing the effects of the curcumin and piperine combination on spontaneous locomotor activity. The clinical relevance of the rodents’ locomotive behaviors to the CNS side effects in humans has previously been established. For example, impaired locomotor activity and rearing in rodents resemble dizziness in humans, and also impaired home cage behaviors are employed as a model for somnolence and fatigue [53]. The LABORAS automatic behavioral analysis system facilitates the successful and precise identification and characterization of each of those rodent behaviors. Hence, it is used as a model to predict the CNS side effects of compounds. We found no effects of the individual curcumin and combination on the spontaneous locomotor activity at their high doses, while individual piperine reduced locomotor activity. The results indicate no potential CNS side effects of the curcumin and piperine combination. Moreover, coadministration of curcumin and piperine could be a potential approach to overcome the locomotor impairment induced by high doses of piperine as it lowers the required therapeutic doses.

## Conclusion

In summary, this study demonstrates that the combination of curcumin and piperine acts synergistically in mouse models of pain without showing any potential CNS side effects. The information on curcumin and piperine interaction in mouse models of pain will perhaps give clinical implications and could be further used to treat patients with pain.

## Data Availability

Data will be made available upon request. Contact pasarapa.c@chula.ac.th.

## Abbreviations

ANOVA: Analysis of variance
CMC: Carboxymethyl cellulose
CNS: Central nervous system
Cur: Curcumin
Cur + Pip: Curcumin piperine combination
ED_50 add_: Theoretical ED_50_ dose
ED_50 exp_: Experimental ED_50_ dose
GABA: Gamma-aminobutyric acid
GRAS: Generally recognized as safe
IL-6: Interleukin-6
LABORAS: Laboratory animal behavior observation registration and analysis system
MMP13: Matrix metallopeptidase 13
MPE: Maximum possible effect
PGE_2_: Prostaglandin E2
Pip: Piperine
TRPA1: Transient receptor potential ankyrin 1
TRPV1: Transient receptor potential vanilloid 1
US FDA: United States Food and Drug Administration
γ: Interaction index
